# Infant Botulism, Israel, 2007–2021

**DOI:** 10.3201/eid2902.220991

**Published:** 2023-02

**Authors:** Bar Goldberg, Dana Danino, Yoel Levinsky, Itzhak Levy, Rachel Straussberg, Halima Dabaja-Younis, Alex Guri, Yotam Almagor, Diana Tasher, Daniel Elad, Zina Baider, Shlomo Blum, Oded Scheuerman

**Affiliations:** Schneider Children’s Medical Center, Petach Tikva, Israel (B. Goldberg, Y. Levinsky, I. Levy, R. Straussberg, O. Scheuerman);; Sackler Faculty of Medicine, Tel Aviv University, Tel Aviv, Israel (B. Goldberg, I. Levy, R. Straussberg, D. Tasher, O. Scheuerman);; Soroka Medical Center, Beer Sheva, Israel (D. Danino);; Faculty of Health Sciences, Ben-Gurion University, Beer Sheva (D. Danino);; Rambam Medical Center, Haifa, Israel (H. Dabaja-Younis);; The Ruth and Bruce Rappaport Faculty of Medicine, Technion, Haifa, Israel (H. Dabaja-Younis);; Kaplan Medical Center, Rehovot, Israel (A. Guri);; Hadassah Faculty of Medicine, The Hebrew University of Jerusalem, Jerusalem (A. Guri);; Meuhedet Healthcare, Jerusalem, Israel (Y. Almagor);; Wolfson Medical Center, Holon, Israel (D. Tasher);; National Reference Laboratory for Botulism, Kimron Veterinary Institute, Bet Dagan, Israel (D. Elad, Z. Baider, S. Blum)

**Keywords:** infant botulism, pediatrics, epidemiology, honey, botulism neurotoxin, Clostridium botulinum, foodborne diseases, food safety, bacteria, Israel

## Abstract

Infant botulism (IB) is an intestinal toxemia that manifests as descending paralysis, constipation, and, in some cases, respiratory failure. Laboratory-confirmed IB cases are rare, and recent data in Israel are lacking. We conducted a national multicenter retrospective study of laboratory-confirmed IB cases reported in Israel during 2007–2021. A total of 8 cases were reported during the study period. During 2019–2021, incidence may have increased because of a cluster of 5 cases. Infant median age for diagnosis was 6.5 months, older than previously reported (3 months). Most cases occurred during March–July. Honey consumption was reported in 1 case, and possible environmental risk factors (living nearby rural or construction areas, dust exposure, and having a father who works as a farmer) were reported in 6 cases. Although IB is rare, its incidence in Israel may have increased over recent years, and its epidemiology and risk factors differ from cases reported previously in Israel.

## Abstract 2

cases indicate a stronger link to soil transmission, possible seasonal variation, and a milder course of disease.

*Clostridium botulinum* is a gram-positive, rod-shaped, spore-forming, obligate anaerobic bacterium. It is ubiquitous in the environment, such as soil and marine sediment, and can be easily isolated from the surfaces of vegetables, fruits, and seafood. Botulinum neurotoxins (BoNTs) secreted by *C. botulinum* bacteria are among the most potent toxins in nature. BoNTs target motor neurons, and block the cholinergic neuromuscular innervation of striated and smooth muscles in multiple tissues. BoNTs are classified into 7 antigenic serotypes (A to G). Types A, B, and, rarely, E and F, are linked to infant botulism (IB). IB can occur when an infant ingests *C. botulinum* spores because of exposure to contaminated soil or agricultural products, notably honey, when the bacteria develop and release BoNTs into the intestine (*1*,*2*).

IB is a rare disease with a peak incidence among infants 2–8 months of age. IB is classically described as a flaccid descending symmetric paralysis, and recovery can take several weeks (*3*). The disease manifests in a wide clinical spectrum, from mild symptoms to life-threatening conditions (*3*–*8*), and often leads to a late diagnosis (*9*,*10*). The standard and the most sensitive and specific in vivo method used to confirm IB is by mouse lethality bioassay (MLB) (*7*). Treatment includes monitoring, supportive management, and administration of antitoxin (*11*–*13*). In the United States, the mortality rate among hospitalized infants is ≈1% (*14*).

Since 1976, at least 3,350 cases of IB have been reported worldwide, 90% of them in the United States, the highest reported incidence in California. Many cases probably are unrecognized or unreported (*15*–*18*). The average incidence in the United States is 2.1 cases/100,000 live births (*15*), corresponding to ≈75–100 cases yearly (*7*).

Israel is a developed country with a high-quality and universally available healthcare system, and botulism diagnosis is conducted in a single centralized reference laboratory. Recent data on IB in Israel are lacking. The main goals of our study were to evaluate the current incidence of infant botulism in Israel and examine national epidemiologic and clinical data from the past 2 decades. The study was approved by the Institutional Review Board at the Rabin Medical Center (approval no. RMC 20–0972).

## Methods

We conducted a retrospective multicenter national cohort study in Israel. Included in this study were all infants 1 week to 1 year of age who had IB diagnosed during 2007–2021 by the National Reference Laboratory for Botulism’s Department of Bacteriology at Kimron Veterinary Institute (Bet Dagan, Israel). In addition, we verified with all pediatric infectious disease specialists in Israel that no other patients had IB diagnosed during the study period. Confirmed case-patients in the study were infants (<1 year of age) with clinical signs and symptoms suggesting IB and confirmed by MLB or mass spectrometric–based endopeptidase (EndoPep-MS) assay for detecting and differentiating BoTN serotypes.

We performed laboratory testing for IB by using standard MLB procedures under Kimron Veterinary Institute institutional approval for ethical use of laboratory animals. In parallel, we injected 0.5 mL of serum intraperitoneally into 2 mice and tested the presence of toxigenic *C. botulinum* bacteria in stool. We inspected mice for symptoms consistent with botulism. When both mice died within 96 hours of inoculation, or showed clear signs consistent with botulism in nonheated samples only, we conducted a neutralization test. We diluted culture supernatant (from stool samples) with A, B, or E antitoxins (provided by the US Centers for Disease Control and Prevention) and injected mice intraperitoneally with 0.5 mL of 1 of the following: native untreated supernatant; supernatant with A, B, or E antitoxin; or native supernatant heated at 80°C for 20 min. Finally, we confirmed botulinum by BoNT typing on the basis of survival and lack of symptoms in mice challenged with 1 of the neutralized samples and the heated samples, and deaths in mice challenged with the remaining native samples.

We used other diagnostic tools, such as an electromyographic test that showed characteristic findings for botulism (e.g., a repetitive stimulation leading to an incremental response), stool PCR, and EndoPep-MS assay (*19*,*20*). The EndoPep-MS assay was conducted by the Israeli Institute for Biologic Research (Ness Ziona, Israel), as described by Rosen et al. (*21*).

We retrieved clinical information about the patients from medical records in 6 hospitals. To evaluate the number of IB cases per live births in Israel, we used data from the Israel Central Bureau of Statistics on live births during the study period (https://www.cbs.gov.il/en/subjects/Pages/Live-Births.aspx). For each case, we collected the following information: demographic data (age, sex, place of residence, hospital where the patient was treated; clinical data (perinatal information, underlying conditions, and breast feeding vs. formula); information on environmental exposures (agricultural occupation of parents or living nearby construction areas), positive toxin test results or clostridial spore presence (as determined by culture or MLB) in honey, formula, soil, and dust from habitant samples; and clinical manifestations. Laboratory results included the toxin type found (A, B, or E), electromyographic features, and blood and cerebrospinal fluid test results. We also collected information on treatment protocols (e.g., use of antibiotics, supportive care only, and administration of antitoxin) and prognosis. 

## Results

We identified 8 IB patients reported in Israel during 2007–2021; of those, 5 were boys and 3 girls. Three stool samples were positive for BoNT A– and 5 for BoNT B–producing *C. botulinum*. Seven patients were born at term and healthy with no underlying conditions. One patient was born at late preterm and had previous known hypotonia but was included in the study because of acute clinical deterioration, a positive BoNT B result, and improvement during sequential neurologic follow-ups.

The incidence of IB in Israel during 2007–2021 was 0.3 cases/100,000 live births (8 cases). A possible increase in IB incidence of IB occurred during 2019–2021 (0.9 cases/100,000 live births [5 cases]), compared with incidence during 1996–2018 (0.14 cases/100,000 live births [5 cases]) (Figure).

**Figure:**
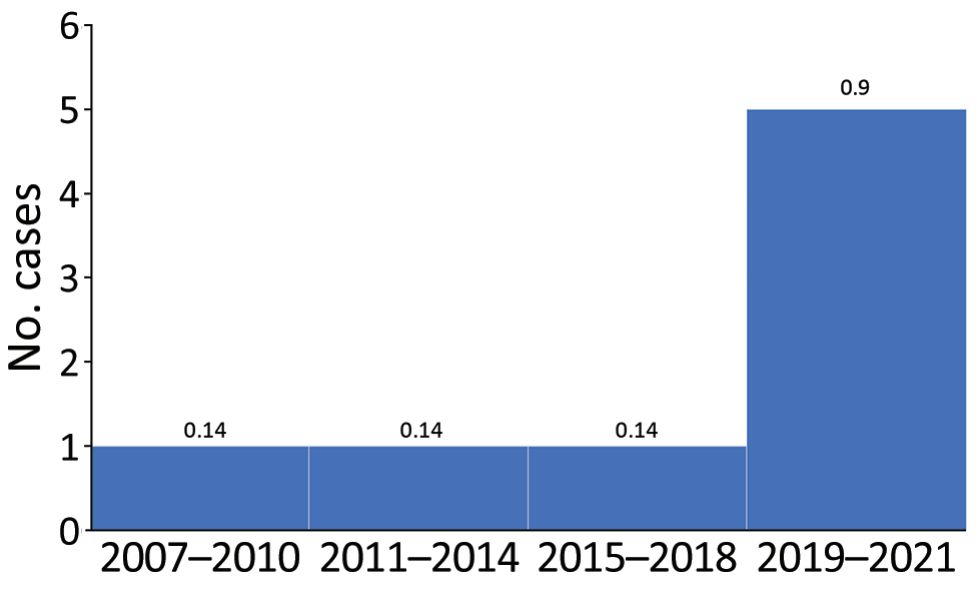
Cases of infant botulism by 3-year period, Israel, 2007–2021. Numbers above bars indicate incidence for that period (no. cases/100,000 population).

The median age for diagnosis in our study was 6.5 months (range 2.5–8 months). The median time to diagnosis (from symptom onset to suspected diagnosis) was 9.5 days (range 4–35 days). In 1 case, which was detected by the Endopep-MS (when MLB results were negative), time to diagnosis was 3 days from symptom onset.

The demographic and geographic data indicated that no geographic cluster occurred and that the cases were evenly distributed across Israel (Table 1). Similarly, ethnicity and socioeconomic characteristics of IB patients were diverse. Two of the case-patients resided in a rural area (1 in a temporary shed, 1 in a private house). The others resided in urban areas.

**Table 1:** Demographic and clinical characteristics of infant botulism patients, Israel, 2007–2021

Patient no.	Age, mo/sex	Ethnicity	Term or preterm	Birthweight, kg	No. siblings	Region	Location type	Form of housing
1	2.5/F	Bedouin	Term	3.1	3	Negev (South)	Rural	Temporary shed
2	4/M	Jewish	Term	3.1	3	Negev (South)	Urban	Apartment building
3	6/M	Jewish	Term	3.3	1	Center	Urban	Apartment building
4	8/F	Jewish	Late preterm	1.9	1	Center	Rural	Private house
5	7/M	Jewish	Term	3.2	1	Center	Urban	Private house
6	8/M	Jewish	Term	2.9	0	Galil (North)	Urban	Apartment building
7	5/F	Jewish	Term	2.8	1	Center	Urban	Apartment building
8	7/M	Jewish	Term	3.6	4	Center	Urban	Apartment building

### Potential Risk Factors

We found several potential risk factors and hazardous exposures. Most (7/8) cases occurred during March–July (spring and summer), and the last case was in a patient from the Negev Desert, characterized by a hot and dry climate year-round. Three infants lived near construction areas and had a history of traveling outside near the construction sites a few days before symptoms began. Two case-patients had a history of being given a homeopathic or plant-based traditional medicine (yannsoon [anise leaves], a common traditional medicine for infantile colic among Bedouin populations, and a nonrecognized mixture). Two patients were breastfed only. One infant was exposed to the contents of a vacuum cleaner. The father of 1 patient was an agriculturist and was therefore exposed to soilborne pathogens. In 1 case, a sample taken from a honey cake that the infant had eaten tested positive by culture and MLB (raw honey was poured over the topping of the cake after baking). No potential risk factors were found for 1 of the patients.

### Clinical Features, Diagnosis, and Prognosis

The most common clinical signs and symptoms were hypotonia, poor feeding, and weak cry (Table 2). The classical descending paralysis was observed in 6/8 infants. Other symptoms, such as mydriasis, facial nerve palsy, hoarseness, and drooling, were less common.

**Table 2:** Clinical features, diagnosis, and course of infant botulism patients, Israel, 2007–2021*

Category	Value
Clinical feature, no. positive/no. tested	
Respiratory distress	3/8
Ptosis	6/8
Facialis	1/8
Poor feeding	7/8
Descending paralysis	6/8
Depressed tendon reflexes	4/6
Hypotonia	8/8
Constipation	7/8
Hoarseness	3/8
Aspiration or decreased gag reflex	4/8
Weak cry	7/8
Lack of smile	4/8
Drooling	1/8
Mydriasis	2/8
Diagnostic tool used, no. positive/no. tested	
Electromyographic test	3/4
Toxin A	3/8
Toxin B	5/8
Stool PCR	2/2
EndoPep-MS	2/2
Mouse lethality bioassay	7/8
Course of illness, d, median (range)	
Time to resolution	75 (16–180)
Duration of nasogastric tube support†	11 (10–27)
Duration of intubation‡	11.5 (2–21)
Duration of ICU stay§	10.5 (11–30)
Duration of hospitalization	16 (11–30)
Time to diagnosis	9.5 (4–35)

*EndoPep-MS, mass spectrometric–based endopeptidase assay for detecting and differentiating botulinum neurotoxin serotypes; ICU, intensive care unit. †5/8 patients were supported by feeding tube. ‡2/8 patients were intubated. §4/8 patients were admitted to ICU.

Five patients were treated with antibiotics at admission: 4 with ceftriaxone (alone or in combination with ampicillin or vancomycin) because of general deterioration and to cover for a possible differential diagnosis of meningitis or epiglottitis (given the drooling observed in the patient) and 1 with azithromycin because the patient’s sister had pertussis. The median length of hospitalization was 13 days for those who were treated with antibiotics versus 16 days for those who were not.

Antitoxin (equine-derived) treatment was given to 4 patients, 3 with trivalent antitoxin and 1 with heptavalent. No adverse reactions were observed. The median length of hospitalization for those who were treated with antitoxin was 17 days versus 13.5 days for those were not.

Only 2 infants were intubated, the youngest (2.5 and 4 months of age) among our study group, and both tested positive for BoNT B. Five of the 8 infants were fed by nasogastric tube for an average duration of 11 days.

### Long-term Prognosis

The long-term prognosis was good: 1 infant with previous hypotonia (before IB diagnosis) had made progress and showed improvement at neurologic follow-ups. One child still had mild fine motor delay at 6 months after diagnosis but recovered completely afterwards. One child had a relative relapse of the symptoms 3 months after initial diagnosis, but no new laboratory evidence indicated presence of spores or toxins, and the child had a complete recovery. No neurologic or other sequelae were found among the other patients, and no deaths were recorded.

## Discussion

In this study, we describe cases of IB in Israel during 2007–2021. We found a possible increase in incidence in the last 3 years of the study period, especially in 2020, compared to the previous 2 decades. We also noted older ages of the infant patients at illness onset compared with those described previously (*4*). Honey consumption was reported in only 1 infant, whereas we observed an apparently stronger link to environmental factors as source of infection.

The age of diagnosis in our study (6.5 months) is much older than that found in other studies. Previous studies found that age at illness onset in 90% of IB cases was 1–6 months (*4*), with a median age of 91 days (*22*,*23*). A reasonable assumption is that around this age, the infant undergoes a sharp transition from breastfeeding-only or formula-fed only to exposure to different foods. All those changes can cause a sharp shift of the bowel microbiome and optimize conditions for colonization and proliferation of *C. botulinum* (*24*). In addition, around this age, the infant is exposed to other external factors, such as daycare environments, solid foods, and other environmental factors, that may expose them to *C. botulinum.*

Previous research has shown that the connection between honey consumption and IB in the United States is considerable; honey consumption accounts for ≈15%–20% of all IB cases, and up to 25% of honey products were found to be positive for *C. botulinum* spores (*3*,*25*,*26*). In Europe, 59% of the IB cases are related to exposure to honey before symptom onset (*18*). In our study, honey consumption was reported in only 1 case (a honey-cake that was later found to be *C. botulinum* contaminated). This modest link is notable, given that people in Israel are known to be avid consumers of both local honey and honey imported from Europe and the United States; further, honey consumption increases during some of the Jewish holidays, around September–October. In fact, we found no cases of IB during those months. Many case series show that IB cases linked to honey consumption were in patients who were positive for BoNT B (*25*). In our study, the infant who consumed honey was also positive for BoNT B. A possible explanation might be an early and mandatory parental education program in outpatient infant clinics in Israel that forbids honey products until the infant is 1 year of age.

We observed a possible link to certain environmental factors, including dust (vacuum cleaner content), having a father who works as a farmer, crowded living spaces, and living near construction sites, all already known as potential risk factors (*8*,*10*,*20*,*25*,*27*). Those cases might indicate that environmental factors are major risk factors and should be investigated thoroughly and carefully.

A possible seasonal relationship emerged from our findings. Most (7/8) of the cases occurred during spring and summer in the Northern Hemisphere (March–July). One case originated from the Negev Desert area, characterized by a year-round hot and dry climate. These findings suggest a possible influence of temperature on IB outbreaks. Supporting evidence of this distinct seasonal incidence was also shown in a study conducted in the US state of Utah, where all cases of IB occurred during March–October and no cases occurred during the winter months (*12*,*22*,*27*). Another possible explanation might be that those are the most convenient months for outdoor activities and traveling, and therefore more exposure to soil and other environmental factors occurs.

Our study demonstrates a possible rise in IB incidence throughout Israel. During 1996–2006 (a 10-year period), only 2 cases of IB were diagnosed (*15*,*28*–*30*), whereas in our study, 8 cases were diagnosed during 2007–2021 (a 15-year period). A possible explanation might be the rapidly increasing number of construction projects, higher population density, and increasing awareness of IB among pediatricians. Still, the national incidence of IB in Israel is relatively low (only ≈0.3 cases/100,000 live births, compared with 1.9–2.1 cases/100,000 live births in the United States [*4**,**15*] and 2.2 cases/100,000 live births in Argentina [*31*]) and is more similar to low-incidence countries such as England (*32*) or Germany (*15*).

We identified a notable cluster of 5 cases during 2019–2021 (0.9 cases/100,000 live births). This possible surge in incidence raises an interesting question about a possible link to the COVID-19 pandemic. Is the increase in IB incidence observed in Israel during 2020 also observed worldwide? No up-to-date data are available to answer this question. This possible surge in Israel might also be attributed to the plausibility of random variations in the occurrence of a rare disease.

Most of the cases in our study were caused by BoNT B (5/8) and the rest by BoNT A (3/8). A similar ratio exists in the United States (62% BoNT and 37% BoNT A), but the ratio is different in the rest of the world, where BoTN A is the more prevalent type (*15*).

The number of infants who required intubation and invasive ventilation in our study was notably lower (2/8) that reported in other case-series reviews, such as one conducted in California, USA (60%–82%) (*22*,*33*,*35*). The 2 infants who were intubated were both positive for BoNT B, which is related to a higher incidence of respiratory failure (*33*). The older age of patients at onset observed in our study led to a milder clinical course and lower intubation rate.

Half of the infants in our study were treated with antitoxin, whereas the rest showed clinical improvement or recovered without antitoxin treatment. Previous reports showed that antitoxin treatment shortened the length of hospitalization from 23 to 13 days (*22*,*33*–*35*). In our study, infants who were treated with antitoxin were hospitalized for 17 days, and those who were not treated were hospitalized for 13.5 days. A possible explanation might be that the infants who received antitoxins had a more severe clinical picture, which necessitated the use of antitoxin but also led to a more complex and prolonged hospitalization. Another possible explanation might be the fact that in Israel the equine-derived botulism antitoxin is still used, whereas in the United States, the common antitoxin administered in recent years is the human botulism immune globulin.

The main strength of our study is its national reach. Because only 1 national laboratory for diagnosis of IB exists, we were able to access all the diagnosed case-patients’ medical records. The main limitation of our study, which derives from the rarity of IB, is the small sample size of only 8 cases. Another limitation is the retrospective nature of our study, which means some details are unrecorded or unknown.

In summary, although IB is still a rare disease, IB incidence may have increased in Israel during our study period, 2007–2021, and especially during the past 3 years of that period. We also found that honey consumption is less prominent as a risk factor compared with environmental exposures. Clinicians should be aware of the possible changing epidemiology and risk factors of this rare but serious disease in Israel.
